# The effects of school bullying victimization on the development of adolescent depression across genders: a hierarchical linear model analysis

**DOI:** 10.1186/s12889-026-27128-3

**Published:** 2026-03-26

**Authors:** Linhui Liu, Jinwei Gai, Mengdan Luo, Yan Zhang, Wenqian Zhao, Qianjin Lou, Minhuan Wu, Ke Zhao, Guohua Zhang, Lan Hong

**Affiliations:** 1 Department of Public Health, Lishui Second People’s Hospital Affiliated to Wenzhou Medical University, Lishui, 323000 China; 2 Department of Clinical Psychology, Lishui Second People’s Hospital Affiliated to Wenzhou Medical University, Lishui, 323000 China; 3 School of Mental Health, Wenzhou Medical University, Wenzhou, 325035 China; 4 Cixi Biomedical Research Institute, Wenzhou Medical University, Ningbo, 315302 China; 5 Second Clinical Medical College, Wenzhou Medical University, Wenzhou, 325035 China; 6 Renji College, Wenzhou Medical University, Wenzhou, 325035 China; 7 Zhejiang Provincial Clinical Research Center for Mental Disorders, The Affiliated Wenzhou Kangning Hospital, Wenzhou Medical University, Wenzhou, 325000 China; 8 Key Research Center of Philosophy and Social Sciences of Zhejiang Province (Institute of Medical Humanities), Wenzhou Medical University, Wenzhou, 325035 China

**Keywords:** Depression, Adolescent, Hierarchical Linear Model, Bullying victimization, Gender

## Abstract

**Background:**

Adolescents are particularly vulnerable to depression. This longitudinal study examines gender differences in the developmental trajectories of depressive symptoms and investigates how school bullying victimization influences these trajectories across genders.

**Method:**

A total of 1,260 adolescents (58.3% female) participated in this study. Bullying victimization and depressive symptoms were measured by self-report at three waves spaced 6 months apart. We used gender as a grouping variable and fit two-level hierarchical linear models (HLM). Level 1 represented within-person change over time, while level 2 modeled between-person factors, including three types of bullying victimization.

**Results:**

HLM results indicated that girls had a higher mean baseline level of depressive symptoms than boys. Depressive symptoms increased over time among boys (*β* = 0.566, *p* = 0.006) but decreased among girls (*β* = −0.618, *p* < 0.001). At baseline, physical bullying (*β* = 2.894, *p* = 0.001) and relational bullying (*β* = 2.336, *p* = 0.002) were associated with higher depressive symptoms in boys. In girls, relational bullying was associated with higher baseline depressive symptoms (*β* = 3.715, *p* < 0.001) and a more negative time slope (*β* = −1.023, *p* = 0.004).

**Conclusion:**

This study revealed the relationships between depression and different forms of bullying between male and female adolescents, providing insights for developing personalized strategies to reduce adolescent depression by considering individual-level factors and longitudinal changes over time.

**Supplementary Information:**

The online version contains supplementary material available at 10.1186/s12889-026-27128-3.

## Introduction

In 2020, the Chinese National Health Commission issued the “Action Plan for Exploring Specialized Services in Depression Prevention and Treatment,” which emphasized the need to enhance early detection and strengthen interventions for key populations [1]. Adolescents are among the key populations that require focused attention. In recent years, the prevalence of depression among adolescents has increased significantly. Globally, about 34% of individuals aged 10–19 are considered at risk for developing depression [2]. According to the *2023 National Depression Blue Book*, about 30% of individuals diagnosed with depression are minors, half of whom are students [3]. Adolescence is a critical stage of development, during which mental health problems can have lifelong negative consequences [4]. Depression impairs academic performance, social relationships, and psychological well-being, and may also increase the risk of self-harm or suicide.

### Genders differences in the development of depression

Research has demonstrated significant gender differences in the developmental trajectory of depressive symptoms. Meta-analyses have shown that adolescent females experience a greater increase in depression symptoms compared to males [2]. Across the lifespan, females are nearly twice as likely as males to develop depression [5]. Western longitudinal studies indicated that depressive symptoms among female adolescents follow an inverted U-shaped trajectory: symptoms rise at around age 13, increase rapidly between 13 and 15, then enter a plateau period, and gradually decline from 18 to 20 [6]. In contrast, male adolescents exhibit consistently lower levels of depressive symptoms, with a generally stable or slightly declining trend [7]. However, Chinese studies have found that depressive symptoms in adolescent males are equal to or even higher than those of females [8], with most males exhibiting a rising trend of depressive symptoms. In contrast, Chinese females have shown an inverted U-shaped trajectory consistent with findings from Western research [9]. Previous studies have largely focused on mean-level comparisons of symptoms and have paid limited attention to the developmental trajectory. This study aims to examine the trajectory of depression symptoms among Chinese adolescents, with a particular focus on gender differences and associated risk factors.

### The relationship between depression and school bullying victimization

Among various risk factors for adolescent depression, school bullying has attracted significant attention as a key form of negative peer interaction. School bullying refers to intentional actions by students to inflict harm on others through physical, verbal, or relational means, resulting in physical injury, psychological distress, or damage to property [10, 11]. According to Olweus, school bullying can be categorized into five types: verbal, physical, relational, sexual, and cyberbullying [11]. Verbal bullying involves insults, teasing, derogatory language, and threats; physical bullying includes pushing, hitting, or damaging others’ property; relational bullying encompasses exclusion, social neglect, and the spreading of harmful rumors [10, 12]. Physical, **v**erbal, and sexual bullying are typically classified as direct forms, whereas relational and cyberbullying are considered as indirect forms of aggression. Among children and adolescents, verbal, physical, and relational bullying are the most prevalent forms [13]. A global survey across 42 countries reported that verbal bullying was the most common form of victimization among adolescents (36.8%), followed by relational (34.9%) and physical bullying (13.3%). In general, boys are more likely to engage in bullying and are commonly both victims and perpetrators of direct forms such as physical or verbal bullying, while girls are more often involved in indirect forms like relational bullying [14].

Bullying increases the risk of depression, primarily due to the disruption of interpersonal relationships. Based on the interpersonal risk model of depression [15], poor relationships with others can trigger, maintain, and worsen depressive symptoms. Likewise, general strain theory also suggests that negative experiences in relationships may be associated with negative emotions. If a person cannot deal with bullying appropriately, they may feel distressed and their risk of depression may increase [16]. According to the 2024 National Report on Adolescent Mental Health and Academic Status, adolescents with negative peer relationships face significantly elevated depression risk [17]. Bullying at school plays a crucial role in causing adverse peer interactions, leading to alienation and social withdrawal in adolescents. Such stressful interpersonal experiences may reinforce negative cognitive patterns and self-perceptions, thereby increasing the risk of depressive symptoms. Research shows that school bullying is responsible for 10.8% of depression cases in teenagers [18], and both cross-sectional and longitudinal studies have shown a positive correlation between bullying victimization and depressive symptoms [19–21]. Being frequently bullied at age eight may be linked to psychiatric disorders in adulthood, even if the person did not show any psychiatric symptoms during childhood [22]. Victims of bullying often suffer from a wide range of psychological and somatic problems, including low self-esteem, depression, anxiety, insecurity, and feelings of isolation [23]. From 1990 to 2019, global DALY rates for anxiety and major depressive disorders attributable to bullying increased by 23.31% and 26.60%, respectively. The increases were significantly greater among females (27.27%/29.07%) than among males (18.88%/23.84%) [24]. Additionally, the association between bullying and suicidal behaviors warrants special attention. Suicide is currently the fourth leading cause of death among young people worldwide, and research has shown that youth suicide rates in non-European regions are not only higher but also characterized by a more notable increase. Among these regions, the United States reports the highest age-standardized suicide rate in males, at 15.5/100,000 population; in the US, the annual increase in suicide rates was 3.8% among males in 2009–2020 and 6.7% among females in 2007–2017 [25]. Notably, involvement in bullying whether as a perpetrator, a victim, or both constitutes a well-established risk factor for suicidality [26]. A meta-analysis has confirmed that adolescents who experience bullying have a 2.23-fold increased risk of suicidal ideation and a 2.55-fold elevated risk of suicide attempts compared with their non-bullied peers [27].

In terms of impact, comparative research has demonstrated that relational bullying is associated with greater emotional distress and depressive symptoms than physical bullying among adolescents [28]. This suggests that different types of bullying may be linked to distinct psychological consequences. Meanwhile, gender moderates the effects of bullying. Females are more likely to experience severe psychological harm, verbal bullying is linked to higher depression risk in females, and relational bullying contributes to both depression and anxiety among them. These effects tend to intensify over time, significantly impairing academic performance and daily functioning [29]. In contrast, males tend to result in more severe consequences by bullying. These findings highlight gender differences in bullying: females primarily experience psychological harm, whereas males are more frequently involved in physical aggression. According to the interpersonal risk model [30], depression arises from three factors: insecure attachment, poor social skills, and excessive reassurance seeking. Such factors increase interpersonal conflict and reduce peer support, thereby exacerbating depressive symptoms [31]. Verbal bullying damages self-esteem, physical bullying arises fear responses and safety concerns, and relational bullying disrupts sense of belonging and social connections. Moreover, cross‑cultural studies suggest that bullying is associated with more severe depressive symptoms among Asian adolescents. These may be explained in emotional expression: Asian cultures often emphasize emotional restraint and self-control, whereas Western cultures encourage expressing emotions directly. Therefore, it is essential to consider cultural context when examining adolescent mental health.

These studies show that bullying is a significant interpersonal risk factor for adolescent depression. Although many studies support this conclusion, for a comprehensive understanding, we still need to explore different dimensions, such as gender differences and the diversity of bullying forms.

### The current study

Numerous studies have investigated the developmental trajectories of adolescent depression and school bullying. However, few studies have simultaneously analyzed the effects of different types of bullying on different genders. Distinguishing school bullying into relational, verbal, and physical categories provides a theoretically grounded framework for analysis. Gender differences exist in both involvement in and sensitivity to different forms of bullying. To better understand the complex interplay of factors influencing adolescent depression, this study constructs a two-level linear model using a large student sample. Accordingly, the study aims to identify individual correlates (exposure to different forms of bullying) and changes over time associated with adolescent depression through multilevel analysis. A Hierarchical Linear Modeling (HLM) is used to examine the longitudinal dynamics of depressive symptoms among adolescents. In the HLM, adolescents’ depression is taken as a dependent variable, the first-level represented within-person change of depression over time. Then, the second-level regression equation is established using the intercept and slope of the first-level model as the dependent variables. Therefore, the HLM is known as the “return of the return”. As a widely used method in longitudinal analysis, HLM can effectively capture both causality among variables and individual-level variation [32].

Based on previous findings, this study investigates the developmental trajectories of depressive symptoms among male and female students, and explores how different forms of bullying contribute to these trajectories across genders. The hypotheses are as follows: (1) females will exhibit higher levels of depressive symptoms and a faster rate of worsening compared with males. (2) relational bullying will be a significant positive predictor of depressive symptoms in females, whereas physical bullying will significantly and positively predict depressive symptoms in males.

## Methods

### Participants and procedures

This study was a school-based prospective longitudinal cohort study conducted in Lishui, China. Using convenience sampling, students were recruited from two junior high schools (grades 7–9) and two senior high schools (grades 10–12). Within each school, intact classes were selected and all eligible students were invited. Students were excluded if they were absent on the survey day, consent was not obtained, or questionnaires were invalid (e.g., extremely short completion time or failed validity checks). Data were collected across three waves: T1 (Mar. 2024; *n* = 2,000), T2 (Sep. 2024; *n* = 1,274), and T3 (Mar. 2025; *n* = 1,150). Attrition from T1 to T2 was mainly due to graduation (*n* = 660). The final analytic sample included participants who completed at least two survey waves (*n* = 1,260), including 525 boys (41.7%) and 735 girls (58.3%). The mean age was 16.87 (SD = 0.85) years. Of all the participants, 145 (11.5%) were single-child, and 300 (23.8%) were left-behind children.

Before questionnaire distribution, written informed consent was obtained from the schools, students, and their parents. All participants were provided with detailed information about the purpose and procedures of the study. They were also assured that their involvement was completely voluntary and anonymous, and that they could withdraw at any time without any consequences. The survey was conducted class by class, with students completing electronic questionnaires in designated school computer labs. This study was conducted in accordance with the principles of the Declaration of Helsinki. The study was approved by the Medical Research Ethics Committee of Lishui Second People’s Hospital Affiliated to Wenzhou Medical University (20240723-08).

### Measures

#### Self‑report demographic survey

Participants filled out the social and demographic section of the survey: gender, age, single-child status (Yes, No) and left-behind children (Yes, No).

#### Depressive symptoms

Adolescents’ depressive symptoms was measured by Center for Epidemiological Studies Depression Scale (CES-D) compiled by Radloff and revised by Chen Zhiyan [33]. This 20-item questionnaire uses a 4-point Likert scale (0 = rarely or none; 3 = most or all of the time), with higher scores indicating more severe depressive symptoms. It has good reliability and validity among Chinese adolescents [34, 35]. The Cronbach’s α coefficients of T1, T2, and T3 were 0.947, 0.946, and 0.948, respectively.

#### School bullying victimization

The “about direct bullying” subscale of the Olweus Bully/Victim Questionnaire (OBVQ) was used to measure adolescents’ school bullying, which was revised by Zhang Wenxin [36]. The 6-item subscale (5-point Likert scale: 0 = none; 4 = several times a week) includes three dimensions: verbal (items 1, 6), relational (items 2, 4), and physical bullying (items 3, 5). Higher scores reflect greater victimization. The OBVQ has good reliability and validity among Chinese adolescents [14, 37]. The Cronbach’s α of the bullying items was good at each time point (T1 α = 0.777; T2 α = 0.755; T3 α = 0.845).

### Data analysis

Descriptive statistics were computed using SPSS 25.0. Two-level multilevel growth models were fitted in M-plus 8.3 using robust maximum likelihood (MLR), with missing outcomes handled via full information maximum likelihood (FIML). First, an unconditional null model (Model 1) decomposed variance in depressive symptoms into within-person and between-person components and yielded the intraclass correlation coefficient (ICC), thereby justifying the multilevel approach. Second, an unconditional linear growth model (Model 2) added TIME at Level 1 to estimate the average trajectory of depressive symptoms and individual variability in change. Third, a quadratic growth model (Model 3) added a TIME² term and was compared with the linear model using − 2 log-likelihood (–2LL), AIC, and BIC. Finally, a conditional full model (Model 4) entered bullying indicators at Level 2 to predict both baseline depressive symptoms (intercept) and change over time (slope). Gender differences were tested in a multigroup framework using Wald tests of cross-group equality constraints. Robustness was examined via sensitivity analyses additionally adjusting for age (Model A) and for age plus single-child and left-behind status (Model B).

## Results

### Attrition analysis

To evaluate potential selection bias due to attrition, we compared baseline characteristics between participants retained at T3 (*n* = 1,150) and those lost to follow-up (*n* = 850). There were no statistically significant differences between the groups at baseline in gender, only-child status, left-behind status, depressive symptoms, or bullying indicators, whereas a significant difference was observed for age (see Table S1).

### Preliminary analysis

Table 1 shows the means, standard deviations, and correlation matrix for the variables. Only gender was found not significantly correlated with relational bullying. All three forms of bullying were positively correlated with depression at each time point.

**Table 1:** The correlations among variables, means, and standard deviations

	M	SD	1	2	3	4	5	6
1 DepressionT1	12.74	10.83	1					
2 DepressionT2	12.82	10.71	0.65^***^	1				
3 DepressionT3	12.28	9.84	0.57^***^	0.64^***^	1			
4 Verbal bullying	0.37	0.84	0.19^***^	0.13^***^	0.08^*^	1		
5 Physical bullying	0.09	0.48	0.22^***^	0.17^***^	0.10^**^	0.52^***^	1	
6 Relational bullying	0.35	0.90	0.31^***^	0.23^***^	0.17^***^	0.58^***^	0.54^***^	1

** *p* < 0.01, *** *p* < 0.001

### HLM model

#### Null model (Models 1)

Repeated measurements (Level 1) were nested within individuals (Level 2). In the equations below, $\:{Y}_{ti}$ denotes the depressive symptom score of individual $\:i\:$at time $\:t$. The Level-1 residual is $\:{\epsilon\:}_{ti}$, and $\:\mu\:$ terms represent between-person random effects.

$$\:{Y}_{ti}\:=\:{\beta\:}_{0i}\:+\:{\epsilon\:}_{ti}$$

$$\:{\beta\:}_{0i}\:=\:{\gamma\:}_{00}\:+\:{\mu\:}_{0i}$$

Model 1 estimates the grand mean ($\:{{\upgamma\:}}_{00}$) and partitions variance into Level-1 within-person residual variance ($\:{{\upsigma\:}}^{2}$) and Level-2 between-person random intercept variance ($\:{{\uptau\:}}_{00}$). As shown in Table S2, the ICC was computed as $\:{{\uptau\:}}_{00}/({{\uptau\:}}_{00}+{{\upsigma\:}}^{2})$, indicating that 63.8% of the total variance was attributable to between-person differences, supporting the use of multilevel modeling.

#### Linear and quadratic growth models (Models 2–3)

We next fitted an unconditional linear growth model (Model 2), where $\:{\beta\:}_{0i}$ is the individual-specific intercept and $\:{\beta\:}_{1i}$ is the individual-specific linear slope. TIME was coded 0–1–2 (T1 = 0, T2 = 1, T3 = 2):

$$\begin{array}{c} {Y}_{ti}={\beta\:}_{0i}+{\beta\:}_{1i}\left(TIM{E}_{ti}\right)+{\epsilon\:}_{ti}\\ {\beta\:}_{0i}={\gamma\:}_{00}+{\mu\:}_{0i}\\ {\beta\:}_{1i}={\gamma\:}_{10}+{\mu\:}_{1i} \end{array}$$

To test potential non-linearity, we added a quadratic term to form the quadratic growth model (Model 3):

$$\begin{array}{l} {Y}_{ti}={\beta\:}_{0i}+{\beta\:}_{1i}\left(TIM{E}_{ti}\right)+{\beta\:}_{2i}\left(TIM{E}_{ti}^{2}\right)+{\epsilon\:}_{ti} \\ {\beta\:}_{2i}={\gamma\:}_{20}+{\mu\:}_{2i} \end{array}$$

Model 3 was compared with Model 2 using − 2LL, AIC, and BIC (Table 2). Because the quadratic model did not improve information criteria (AIC/BIC increased), the linear time specification was retained.

**Table 2:** Model fit indices for two-level multilevel models

Model	Model description	–2LL	AIC	BIC
0	Null model	26347.566	26353.566	26372.187
1	Linear growth model	26348.072	26358.072	26389.107
2	Quadratic growth model	26347.748	26361.749	26405.199
3	Full model	26202.750	26226.750	26301.236

Smaller − 2LL/AIC/BIC indicates better relative fit. Compared with the linear model, adding the quadratic term did not improve information criteria (AIC/BIC increased); therefore, the linear time specification was retained in subsequent analyses

#### Full model (Model 4)

Based on the growth model comparison above, the linear time specification was used in the conditional full model. Bullying predictors were entered at Level 2 (between-person; grand-mean centered) to predict both baseline depressive symptoms (intercept) and change over time (slope):

$$\begin{aligned} &{Y}_{ti}={\beta\:}_{0i}+{\beta\:}_{1i}\left(TIM{E}_{ti}\right)+{\epsilon\:}_{ti}\\& {\beta\:}_{0i}={\gamma\:}_{00}+{\gamma\:}_{01}({verbal\:bullying}{)}_{i}+{\gamma\:}_{02}({physical\:bullying}{)}_{i}\\& \quad \quad \quad +{\gamma\:}_{03}({relational\:bullying}{)}_{i}+{\mu\:}_{0i}\\& {\beta\:}_{1i}={\gamma\:}_{10}+{\gamma\:}_{11}({verbal\:bullying}{)}_{i}+{\gamma\:}_{12}({physical\:bullying}{)}_{i}\\& \quad \quad \quad +{\gamma\:}_{13}({relational\:bullying}{)}_{i}+{\mu\:}_{1i} \end{aligned}$$

As shown in Table 3, at the between-person level, boys who reported higher physical bullying (*β* = 2.894, *p* = 0.001) and relational bullying (*β* = 2.336, *p* = 0.002) had higher baseline depressive symptoms. Among girls, only relational bullying was associated with higher baseline depressive symptoms (*β* = 3.715, *p* < 0.001). Regarding change over time, boys showed a positive mean time slope (*β* = 0.566, *p* = 0.006), indicating an overall increase in depressive symptoms across waves, whereas girls showed a negative mean slope (*β* = −0.618, *p* < 0.001), indicating an overall decrease. Among girls, relational bullying significantly predicted the time slope (*β* = −1.023, *p* = 0.004), suggesting that higher relational bullying was associated with a more negative slope (i.e., a steeper decline) over time. Notably, verbal bullying was not significant at any level of the model.

**Table 3:** Analysis results of the complete model for male and female groups

Fixed effects	Male Estimate (SE)	*p*	Female Estimate (SE)	*p*
Intercept estimates				
Intercept mean	10.951 (0.419)	< 0.001	14.754 (0.383)	< 0.001
Verbal bullying	0.303 (0.597)	0.612	0.482 (0.907)	0.595
Physical bullying	2.894 (0.893)	0.001	−0.598 (1.610)	0.710
Relational bullying	2.336 (0.740)	0.002	3.715 (0.672)	< 0.001
Slope estimates				
Slope mean	0.566 (0.206)	0.006	−0.618 (0.174)	< 0.001
Verbal bullying	−0.118 (0.268)	0.660	0.032 (0.424)	0.940
Physical bullying	−1.050 (0.541)	0.052	0.516 (0.763)	0.499
Relational bullying	−0.558 (0.350)	0.110	−1.023 (0.350)	0.004
Random parameter				
Level-1 residual variance: σ²	38.702 (4.054)	< 0.001	38.900 (3.111)	< 0.001
Level-2 intercept variance: τ_00_	59.233 (7.619)	< 0.001	75.221 (7.246)	< 0.001
Level-2 slope variance: τ_11_	1.767 (2.291)	0.440	1.236 (1.968)	0.530
Cov (intercept, slope)	0.154 (3.476)	0.965	−4.159 (2.595)	0.109

TIME was coded 0 (T1), 1 (T2), and 2 (T3). The TIME slope indicates change per wave. Bullying predictors were grand-mean centered at Level 2

Wald tests indicated significant gender differences (*p* < 0.001). Bullying effects were essentially unchanged after covariate adjustment (Table S3), and key findings remained significant after BH–FDR correction (Table S4).

## Discussion

Using a HLM approach, this study longitudinally examined how physical and relational bullying influence the developmental trajectories of depressive symptoms in male and female adolescents. Our findings revealed the gender-specific patterns of depressive symptom changes and the unique roles of different bullying forms, providing new insights into the complex interplay between school bullying, gender, and adolescent depression. These findings underscore the need for targeted interventions that consider both gender differences and specific bullying forms to address adolescent depression effectively.

### Gender differences in the development of depression among adolescents

The results showed that the baseline level (intercept) of depression in girls was higher than that in boys. Boys exhibited an upward slope in depressive symptoms across the study period, in contrast, girls demonstrated a decreasing slope. These results are partially consistent with the research hypotheses. First, regarding the intercept, girls reported significantly higher baseline levels of depressive symptoms than boys. This finding is consistent with extensive literature documenting greater depression prevalence and severity in females starting in early adolescence [38]. This difference can be explained by response style theory, which posits that females are more likely than males to engage in ruminative coping and negative self-appraisal when facing adversity, thereby increasing their vulnerability to depressive moods [39]. In contrast, males are more likely to adopt active coping strategies, particularly problem-solving approaches, potentially reducing their susceptibility to depression. Biological evidence indicates that men have a 52% higher 5-hydroxytryptamine synthesis rate than women, which may help explain their lower rates of moderate-to-severe depression [40].

Regarding the slope, the trajectories of depressive symptoms over time diverged by gender: boys exhibited an increasing trend (positive slope), whereas girls showed a decreasing trend (negative slope) across the study period. This result is inconsistent with the findings of Hankin et al. [41]. However, some studies support our findings [9]. Researchers mostly agree on how girls’ depression changes in early and middle adolescence, but for boys the pattern is still debated. Studies have found that eighth-grade girls experience higher levels of depressive symptoms that decline across the transition to high school, while boys show lower levels in eighth grade that increase across this transition [42]. This pattern may be related to girls reporting higher friendship quality during the middle school transition and being more likely than boys to participate in high school activities [43]. Indeed, a recent meta-analysis of European-based studies reported that among low/moderate risk-of-bias studies, the pooled estimate for increased depressive symptoms was higher for adolescent boys (aged 16–19 years) than for girls [44]. The authors posit that this trend may reflect greater willingness among boys to report depressive symptoms, not a true increase in prevalence. Such shifts may be associated with growing societal acceptance of male emotional expression and reduced pressures to conform to traditional gender roles (e.g., act tough rather than vulnerable) in recent years. These psychosocial differences underscore the importance of examining the developmental trajectories of depression in males and females separately in order to better understand gender-specific risk and protective factors.

### Effect of bullying on depressive symptoms in females

Consistent with prior studies [14], relational bullying significantly predicts girls’ baseline depression level; the more severe relational bullying is, the higher their initial depression level. Compared with boys, evidence indicates that girls exhibit greater vulnerability to depressive symptoms, especially under repeated bullying victimization [45]. Girls are more likely than boys to experience depression, suicidal thoughts, and suicide plans, and this gender difference is widening [46]. This may be linked to girls’ heightened sensitivity to stressful interpersonal events [47], a pattern that predicts greater relational aggression in girls than in boys [48]. This pattern is consistent with the interpersonal risk model.

Furthermore, in the girl group, relational bullying (BULLR) predicted both higher baseline depressive symptoms and a more negative slope of depressive change (i.e., a faster decrease during follow-up). This high initial level–faster decline pattern should not be interpreted as indicating a protective effect of relational bullying against depression in girls. Instead, it is more likely driven by three interrelated mechanisms. First, individuals with higher baseline depression are more prone to regression to the mean in repeated measurements: extreme initial scores tend to move toward the population mean over time, resulting in a larger reduction and thus a more negative slope [49]. Second, girls exposed to high relational bullying often experience elevated emotional distress and interpersonal threat at T1, leading to higher baseline depressive scores. However, during follow-up, they may more actively initiate or obtain coping and support resources, such as seeking peer or family support, accessing school psychological services, and adjusting their social circles and interpersonal boundaries, which in turn leads to a more pronounced reduction in depressive symptoms. Previous longitudinal studies have shown that relational victimization is consistently associated with depressive symptoms, and factors such as social support shape how this association unfolds over time [50]. Third, there may be dynamic reciprocal effects and co-development between depression and peer bullying. That is, the level of bullying itself may increase or decrease at T2 and T3, with depressive symptoms changing accordingly. Studies using latent change-score approach have demonstrated that peer victimization and depression show developmental interplay in multi-wave longitudinal designs, and their trajectories evolve together over time [51]. Accordingly, we interpret this slope effect as indicating that high relational bullying identifies a subgroup with greater initial depressive risk and subsequent changes in risk exposure and adaptation processes. Future studies could further treat bullying as a time-varying predictor (or adopt parallel process or cross-lagged models) to examine the synchrony and directionality of “changes in bullying–changes in depression.”

### Effect of bullying on depressive symptoms in males

Physical and relational bullying significantly predict boys’ baseline level of depression. Boys are more likely to bully others physically and also to be bullied physically [14, 52]. In traditional gender roles, physical bullying is perceived as masculine behavior that gives boys more power and dominance. Such role expectations may prompt boys to adopt more direct or aggressive strategies including physical and verbal bullying to resolve conflicts or competition and to express frustration impulsively and confrontationally [53], consistent with social norms [52]. When aggression is framed as a masculine trait, boys are more likely to engage in peer victimization and underestimate the consequences [54]. Second, when boys experience physical bullying, the vulnerability displayed conflicts sharply with the “mask of masculinity” dictated by the “Boy Code”. This social stigma may coexist with a self-identity crisis, damaging self-worth, thereby increasing “silent” depression [55].

Adolescence is a critical developmental stage characterized by heightened challenges and vulnerabilities in peer relationships, identity formation, and emotional health [56]. Social relationships form the cornerstone of psychological well-being, but relational bullying damages this foundation. For both males and females, exclusion or isolation impacts the sense of belonging, leading to emotional problems such as depression and anxiety [57]. When an individual holds a negative view of themselves, they are more likely to experience negative emotions. Specifically, peer victimization, as a negative life event, is associated with the development of negative self-attitudes in adolescents, along with negative emotions and elevated depression risk [58]. Stress from adverse experiences and interpersonal conflict may be associated with negative emotions, and thus with elevated depression risk [16].

Furthermore, none of the three forms of bullying produced statistically significant changes in boys’ developmental trajectories of depressive symptoms, consistent with findings by Pedditzi and Lucarelli [59]. Some possible reasons are as follows: First, male depression often manifests as externalizing behaviors, which include substance abuse, aggressive or impulsive behavior, rather than typical internalizing symptoms such as negative mood or self-blame [53]. Direct bullying may thus be associated with a “defensive response” in boys, prompting stress release through behaviors such as revenge or excessive mobile phone use, rather than emotional internalization. Magovcevic et al. found that men following hegemonic masculine norms are more likely to exhibit externalizing symptoms after stressful life events [60]. This behavioral mode of emotion expression can hide the direct association between bullying and depression. Second, masculine norms can prevent help-seeking among boys with depression [61]. After being bullied, boys may suppress depressive feelings to avoid being perceived as weak, or minimize emotional exposure through denial or rationalization (such as “It’s nothing”). This cognition makes the identification of depressive symptoms complicated. Third, most existing depression assessment tools are based on symptoms more typical in women, such as low mood. The effects of bullying on boys may manifest as non-typical symptoms, which complicates the detection of statistical associations with depression. Studies using gender-sensitive tools such as the Goleman Male Depression Scale (GMDS), the Male Depression Risk Scale-22 (MDRS-22), and the Gender-Sensitive Depression Screening Scale (GSDS) have revealed similarly depression prevalence in both genders [62]. These tools capture male-typical symptoms such as anger, aggression, distraction, avoidance, emotional suppression, irritability, substance abuse, and risk behavior, suggesting that traditional tools based on mood may underestimate depression in males.

Notably, in the present study, verbal bullying was not statistically associated with either baseline depressive symptoms or the rate of change in depressive symptoms for boys or girls. First, verbal bullying may be relatively frequent and normalized in school settings, and some adolescents may interpret behaviors such as teasing or name-calling as “joking” or ordinary peer conflict [63], which may reduce its discriminability with respect to depressive symptoms and make it less likely to be recognized and reported. Second, identifying verbal bullying relies heavily on subjective thresholds and contextual interpretation (e.g., peer interaction styles, relational closeness, and cultural norms) [64]; thus, self-report assessments may be more susceptible to variability in interpretation and potential misclassification, which could attenuate observable associations. In sum, these factors may have reduced the unique, reliably measured signal of verbal bullying, making its associations with both baseline depressive symptoms and change over time harder to detect.

### Practical implications and applications

In 2016, China issued the *Guiding Opinions on Preventing and Combating Bullying and Violence among Primary and Secondary School Students*, showing strong national attention to school bullying governance. Based on gender differences identified in this study, targeted interventions can improve the effectiveness of anti‑bullying practices. Schools should establish a comprehensive anti‑bullying framework, including formal policies, enhanced monitoring, standardized reporting systems, and holistic education on mental health and social skills. Gender‑specific interventions are strongly recommended due to distinct patterns in bullying involvement and depressive risks.

For girls, who are more vulnerable to relational bullying and higher baseline depressive symptoms, junior high schools should conduct routine depression screening to identify at‑risk students early. Schools can provide psychological counseling and family intervention, and teach students to recognize covert bullying such as exclusion, rumor‑spreading, and social isolation. Students should also learn adaptive strategies including setting boundaries, seeking help, and building positive peer relationships. For boys, who are more involved in physical bullying and aggressive or withdrawn bully/victim profiles, interventions should focus on early identification of problematic behaviors. Schools should enforce a zero‑tolerance policy for physical bullying, train students in nonviolent conflict resolution, and maintain accessible reporting channels and support groups for victims. Four general principles support broad application of these findings: (1) timely and standardized intervention for all forms of bullying; (2) joint teacher and parent monitoring of subtle harmful behaviors such as mockery, belittlement, and abusive nicknames; (3) early detection and rapid response to prevent conflict escalation; (4) regular depression screening and sustained psychological support for victims. These practical guidelines help translate research results into actionable strategies for educators, parents, and policymakers, facilitating effective bullying prevention and adolescent mental health protection.

### Limitations

The limitations of this study are as follows: (1) all variables were measured using self-report questionnaires, which may be associated with potential reporting bias and social desirability bias. As a result, the prevalence of bullying victimization and depressive symptoms might be underestimated. Future studies are encouraged to combine multiple informants, such as parent ratings, teacher ratings, or peer nominations, to improve the objectivity and reliability of measurements; (2) given the study design, school bullying victimization was only assessed at baseline. This cross-sectional measurement of bullying cannot capture its dynamic and cumulative effects on the development of depression over time. Therefore, future longitudinal studies should measure bullying victimization repeatedly across time points to explore its time-varying impacts on adolescent depressive symptoms; (3) this study also faces several methodological constraints. Participant attrition during the follow-up period may affect the representativeness of the sample, and the lack of adjustment for potential confounding variables may reduce the accuracy of the observed associations. In addition, the clustering effect of students within classes or schools was not fully considered in the analytical model. Future longitudinal research may address these issues by controlling for sample attrition, adjusting for relevant covariates, and adopting appropriate statistical strategies to account for clustered data structure, thereby enhancing the robustness and generalizability of the results. (4) although baseline depression and bullying measures were comparable between retained and lost participants, attrition was associated with graduation, which may limit generalizability to older students.

## Conclusions

This study employs HLM to examine the longitudinal relationships between adolescent depressive symptoms, school bullying, and gender. This study draws the following conclusions: (1) female adolescents start with higher depressive symptoms than males; (2) depression levels increased among males but decreased among females; (3) physical and relational bullying were significantly associated with higher baseline depression among males; (4) relational bullying was significantly associated with higher baseline depression among females, and had a negative impact on depression trajectories, which was associated with intensified symptoms or delayed recovery.

## Supplementary Information

Supplementary Material 1.

## Data Availability

The data used and analyzed during the study are available from the corresponding author if the request is reasonable.
