# Supplementary material for: The effects of school bullying victimization on the development of adolescent depression across genders: a hierarchical linear model analysis
Source: BMC Public Health. 2026 Mar 26;26:1455. doi: 10.1186/s12889-026-27128-3 (PMC13141561; doi:10.1186/s12889-026-27128-3)
Supplement: Supplementary file 1 — Supplementary Material 1. [file 12889_2026_27128_MOESM1_ESM.pdf]

Table S1. Attrition analysis comparing retained vs. lost participants at baseline (T1)

Baseline variables	Retained (n = 1150)	Lost (n = 850)	*p* value	Effect size
Gender (n, %)				
Male	481 (41.8)	366 (43.1)	0.581	Cramér's V = 0.012
Female	669 (58.2)	484 (56.9)		
Single-child (n, %)				
Yes	129 (11.2)	118 (13.9)	0.073	Cramér's V = 0.040
No	1021 (88.8)	732 (86.1)		
Left-behind (n, %)				
Yes	269 (23.4)	207 (24.4)	0.618	Cramér's V = 0.011
No	881 (76.6)	643 (75.6)		
Age (mean±SD)	16.86 ± 0.85	18.07 ± 0.91	< 0.001	Cohen's d = −1.385
Depressive symptoms (mean±SD)	12.72 ± 10.80	13.59 ± 11.30	0.084	Cohen's d = −0.079
Verbal bullying (mean±SD)	0.35 ± 0.81	0.38 ± 0.91	0.471	Cohen's d = −0.033
Physical bullying (mean±SD)	0.09 ± 0.48	0.08 ± 0.48	0.638	Cohen's d = 0.021
Relational bullying (mean±SD)	0.34 ± 0.89	0.36 ± 0.99	0.691	Cohen's d = −0.018

Table S2. Null model results.

Fixed Parameter	Estimate	SE	*p*
Mean (γ_{00})	13.06	0.26	**< .001**
Level-1 (within-person) variance (σ^2)	41.53	1.94	**< .001**
Level-2 (between-person) variance (τ_{00})	73.07	4.29	**< .001**
ICC	0.638		

Note. ICC = τ_{00} / (τ_{00} + σ^2). σ^2 is the Level-1 residual variance; τ00 is the Level-2 random-intercept variance.

Table S3. Robustness checks: bullying subtype effects on depressive symptom intercept and time slope with covariate adjustment (Models A–B).

**Parameter**	**Primary Model** *β* **(SE),** *p*	**Model A** *β* **(SE),** *p*	**Model B** *β* **(SE),** *p*
**Male**			
**Intercept**			
Verbal bullying	0.303 (0.597), 0.612	0.267 (0.600), 0.656	0.283 (0.608), 0.642
Physical bullying	2.894 (0.893), 0.001	2.905 (0.892), 0.001	2.857 (0.898), 0.001
Relational bullying	2.336 (0.740), 0.002	2.339 (0.741), 0.002	2.391 (0.749), 0.001
**Slope**			
Verbal bullying	−0.118 (0.268), 0.660	−0.107 (0.268), 0.691	−0.092 (0.258), 0.722
Physical bullying	−1.050 (0.541), 0.052	−1.055 (0.542), 0.051	−1.048 (0.542), 0.053
Relational bullying	−0.558 (0.350), 0.110	−0.558 (0.353), 0.114	−0.599 (0.355), 0.091
**Female**			
**Intercept**			
Verbal bullying	0.482 (0.907), 0.595	0.496 (0.907), 0.584	0.444 (0.910), 0.626
Physical bullying	−0.598 (1.610), 0.710	−0.576 (1.596), 0.718	−0.706 (1.573), 0.653
Relational bullying	3.715 (0.672), <0.001	3.659 (0.673), <0.001	3.714 (0.667), <0.001
**Slope**			
Verbal bullying	0.032 (0.424), 0.940	0.021 (0.425), 0.960	0.097 (0.426), 0.820
Physical bullying	0.516 (0.763), 0.499	0.498 (0.779), 0.522	0.538 (0.775), 0.487
Relational bullying	−1.023 (0.350), 0.004	−0.992 (0.353), 0.005	−1.041 (0.348), 0.003

Note. Primary Model = bullying subtype predictors only (no additional covariates). Model A additionally adjusted for age. Model B additionally adjusted for age, single-child status, and left-behind status.

Table S4. Bullying subtype effects on depressive symptom intercept and time slope in boys and girls, with BH–FDR adjustment.

Outcome	Predictor	Boys *β* (SE)	*p*	q	Girls *β* (SE)	*p*	q
**Intercept**	Verbal bullying	0.303 (0.597)	0.612	0.775	0.482 (0.907)	0.595	0.775
	Physical bullying	2.894 (0.893)	0.001	0.006	−0.598 (1.610)	0.710	0.775
	Relational bullying	2.336 (0.740)	0.002	0.008	3.715 (0.672)	<0.001	0.006
**Slope (S)**	Verbal bullying	−0.118 (0.268)	0.660	0.775	0.032 (0.424)	0.940	0.940
	Physical bullying	−1.050 (0.541)	0.052	0.125	0.516 (0.763)	0.499	0.775
	Relational bullying	−0.558 (0.350)	0.110	0.220	−1.023 (0.350)	0.004	0.012

Note. q-values are BH–FDR adjusted across 12 bullying-related coefficients (3 subtypes × 2 outcomes × 2 genders). $q < .05$ indicates significance after FDR control.
